# Complete genome sequence of *Citroniella saccharovorans* DSM 29873, isolated from human fecal sample

**DOI:** 10.1128/mra.00015-24

**Published:** 2024-03-11

**Authors:** George Cheng, Maria Westerholm, Anna Schnürer

**Affiliations:** 1 Department of Molecular Sciences, Biocenter Swedish University of Agricultural Sciences, Uppsala, Sweden; Wellesley College, Wellesley, Massachusetts, USA

**Keywords:** human microbiome, fecal organisms, *Citroniella saccharovorans*, Nanopore

## Abstract

A complete genome was recovered from *Citroniella saccharovorans,* strain DSM 29873, using Oxford Nanopore Technologies. The genome assembly contains 1,413,868 bp with 30.23% G+C content. The species belongs to the family *Peptoniphilaceae* and, as of yet, is the only cultivated representative of the genus *Citroniella*.

## ANNOUNCEMENT

*Citroniella saccharovorans* (DSM 29873) was isolated from a fecal sample from a traditional native on the southern coast of Peru (1). *C. saccharovorans* grows under strict anaerobic conditions at optimum 30°C and pH 6.5–7.3, utilizing different sugars, organic acids, and amino acids for growth, with production of acetate and methyl succinate (1). This complete genome will provide possibilities to recover information on genes and potential metabolic pathways used by this species, representing the only isolate of the genus *Citroniella*. This strain was initially enriched anaerobically by inoculation of a fecal sample in liquid medium 2 (1). For isolation, aliquots of the enrichment were transferred to solid agar plates and colonies were finally picked and sub-cultured on the same medium. Patel et. al. (1) performed phylogenetic analysis of the 16S rRNA gene (NR_173562) using UltraClean Microbial DNA Isolation Kit (MoBio Laboratories) and PCR amplification using the universal primers 8F (5´-AGAGTTTGATCCTGGCTCAG-3´) and 1492R (5´-GGTTACCTTGTTACGACTT-3´) (1).

*C. saccharovorans* was retrieved from DSMZ (DSM 29873) as a lyophilized strain and reconstituted following DSMZ instructions, then streaked onto Columbia Blood Agar (ThermoFisher Scientific Columbia Blood Agar with sheep blood medium, PB5039A) in an anaerobic chamber (Whitley A45 anaerobic workstation) at 37°C. After 5 days, a single colony was used for colony PCR amplification (PuReTaq Read-To-Go PCR beads, Cytiva, UK) using primers 27F (5´-AGAGTTTGATCCTGGCTCAG-3´) and 1492R. The PCR cycle was initiated with denaturing step at 95°C for 15 min, 30 cycles at 95°C, then 55°C and 72°C for 1 min each step, finishing with an extension step at 72°C for 7 min (2). Sanger sequencing of the PCR product resulted in a 1,400 bp fragment, showing 99.42% identity to *C. saccharovorans* strain DSM 29873.

DNA was recovered using the NucleoBond kit (Macharey Nagal) following extraction protocol from Sun et al. (3), with modifications in steps 1–6, replaced with resuspension of colonies from solid medium into 2.5 mL Eppendorf tube with Buffer G3. Oxford Nanopore MinION ligation sequencing kit (SQK-LSK109) and native barcoding expansion (EXP-NBD104) were used for sequencing without shearing and size selection. Flow cell FLO-MIN106D R9v and the software was running MinKNOW (v23.04.6) with GUPPY (v6.5.7) basecalling with High Accuracy.

The genome assembly was completed by Nanozoo GmbH. All tools were used with default parameters unless stated otherwise. Fastq_utils (v0.24.1) (4) and seqkit (v0.13.2) (5) were used for validation and manipulation of fastq files, filtltong (v0.2.0) (6) was used to filter long reads by quality, bbmap (v38.86) (7) was used for read alignment, and Flye (v2.9.1) (8) was used for genome assembly and created consensus sequences with medaka (v1.9.1) (9). Annotation was completed with NCBI prokaryotic genome annotation pipeline (PGAP) (10). Bandage (v0.8.1) (11) was used for visualizing *de novo* assembly graphs. The completeness of the genome was verified with CheckM (v1.1.3) (12). The assembled genome size is 1,953,712 bp with a genome coverage of 40× and a completeness of 98.42% and 0% contamination. The final assembly contains three contigs with GC content of 30.23% and N_50_ of 1,413,868 bp. The annotated genome has 1,939 predicted genes, 1,893 coding sequences (CDSs), 37 tRNA sequences, two 5S, two 16S, and two 23S rRNA genes.

## Data Availability

This Whole Genome Shotgun project has been deposited at DDBJ/ENA/GenBank under the accession JAYKOT000000000. The version described in this paper is the first version, JAYKOT000000000.1. The Nanopore reads can be found under BioProject accession PRJNA1050169.
